# The cardioprotective role of the G protein–coupled receptor FFAR4 in atherosclerosis is independent of macrophage foam cell regulation

**DOI:** 10.1016/j.jbc.2025.108463

**Published:** 2025-03-27

**Authors:** Gage M. Stuttgen, Caroline J. Ring, Vishnu S. Guda, Guadalupe K. Valdivia Esparza, Daisy Sahoo

**Affiliations:** 1 Department of Biochemistry, Medical College of Wisconsin, Milwaukee, Wisconsin, USA; 2 Cardiovascular Center, Medical College of Wisconsin, Milwaukee, Wisconsin, USA; 3 Center for Immunology, Medical College of Wisconsin, Milwaukee, Wisconsin, USA; 4 Department of Medicine, Division of Endocrinology & Molecular Medicine, Medical College of Wisconsin, Milwaukee, Wisconsin, USA

**Keywords:** free fatty acid receptor 4, cholesterol, oxidized low-density lipoprotein, foam cell, cardiovascular disease, atherosclerosis, macrophage

## Abstract

Free fatty acid receptor 4 (FFAR4), also known as G protein–coupled receptor 120, is a long-chain unsaturated fatty acid receptor expressed in multiple tissue types including macrophages. Activation of FFAR4 maintains metabolic homeostasis by regulating adipogenesis, insulin sensitivity, and inflammation. While FFAR4 is best known for its protective role in obesity and diabetes, recent studies have demonstrated that FFAR4 may also prevent the development of atherosclerosis and cardiovascular disease. Given FFAR4’s importance in anti-inflammatory signaling in macrophages, we used peritoneal macrophages from WT and FFAR4 KO (*Ffar4*^*−/−*^*)* mice to test the hypothesis that FFAR4 prevents the development of macrophage foam cell formation. Our data suggest that neither activation of FFAR4 nor deficiency of FFAR4 has any influence on foam cell outcome in oxidized low-density lipoprotein–treated macrophages. These data suggest that FFAR4’s cardioprotective roles in atherosclerosis are independent of the regulation of macrophage foam cell formation.

Cardiovascular disease (CVD) is the leading global cause of death accounting for 13% of the world’s total deaths (1). In the United States alone, 702,880 people died from CVD in 2022 (2). The most common cause of CVD is atherosclerosis, which occurs when plaque accumulates in the arteries and restricts blood flow to the heart. Atherosclerosis is characterized as chronic inflammation initiated by the interaction between modified lipoproteins and monocyte-derived macrophages in the tunica intima of damaged endothelial cells (reviewed in (3, 4, 5)). In atherosclerosis, low-density lipoprotein (LDL) becomes trapped in the intima, where it is subjected to oxidative modification by reactive oxygen species to form proatherogenic oxidized LDL particles (oxLDL) (reviewed in (6, 7)). The binding of oxLDL to endothelial cells triggers the release of cytokines that recruit monocytes to the site of tissue damage (8, 9). Monocytes then differentiate into macrophages that take up oxLDL cholesterol *via* scavenger receptors (10, 11). Continual cycling through this process contributes to macrophage foam cell formation and the buildup of cholesterol and arterial plaques.

Free fatty acid receptors (FFARs) are critical nutrient-sensing receptors that exhibit diverse functions in metabolism, cardiovascular health, and inflammation. Currently, four FFARs have been identified based on their physiological importance and have attracted attention as potential therapeutic targets for metabolic and immune disorders (reviewed in (12, 13, 14, 15)). FFAR4, also known as G protein–coupled receptor 120, is a long-chain unsaturated fatty acid receptor expressed in multiple tissues and cells including macrophages (12, 16). FFAR4 plays protective roles in metabolic disease and dysfunction of FFAR4 has been shown to contribute to insulin resistance and obesity in both humans and mice (17). Recently, FFAR4 has emerged as a new player in CVD (reviewed in (18)). Several *in vitro* studies describe how FFAR4 protects against the initial steps of atherosclerosis by regulating monocyte–endothelial cell interactions and endothelial cell–mediated inflammation (19, 20). Activation of macrophages by FFAR4-specific agonists (GW9508 or TUG-891) decreased secretion of pro-inflammatory cytokines (21, 22) and increased cholesterol efflux (23), while higher levels of FFAR4 expression correlated with increased migration of bone marrow–derived macrophages (24). Additionally, *in vivo* studies have shown that activation of FFAR4 by GW9508 or TUG-891 reduces the size of atherosclerotic plaques in apolipoprotein E KO mice (25, 26). Together, these studies support the idea that FFAR4 plays a cardioprotective role by reducing atherosclerotic plaque accumulation and lesion inflammation.

Uncontrolled uptake of oxLDL cholesterol leads to the formation of lipid-laden macrophage foam cells, a hallmark of atherosclerosis that precedes plaque buildup in the arteries. As such, reducing foam cell formation is a potential strategy to decrease CVD risk and presents an opportunity for targeted therapies. In this study, we designed experiments using primary mouse peritoneal macrophages treated with FFAR4 agonist GW9508 or cpdA, as well as FFAR4-deficient peritoneal macrophages to test the hypothesis that FFAR4 plays a cardioprotective role by preventing macrophage foam cell formation.

## Results

### Incubation of peritoneal macrophages with oxLDL generates foam cells

**Figure 1:**
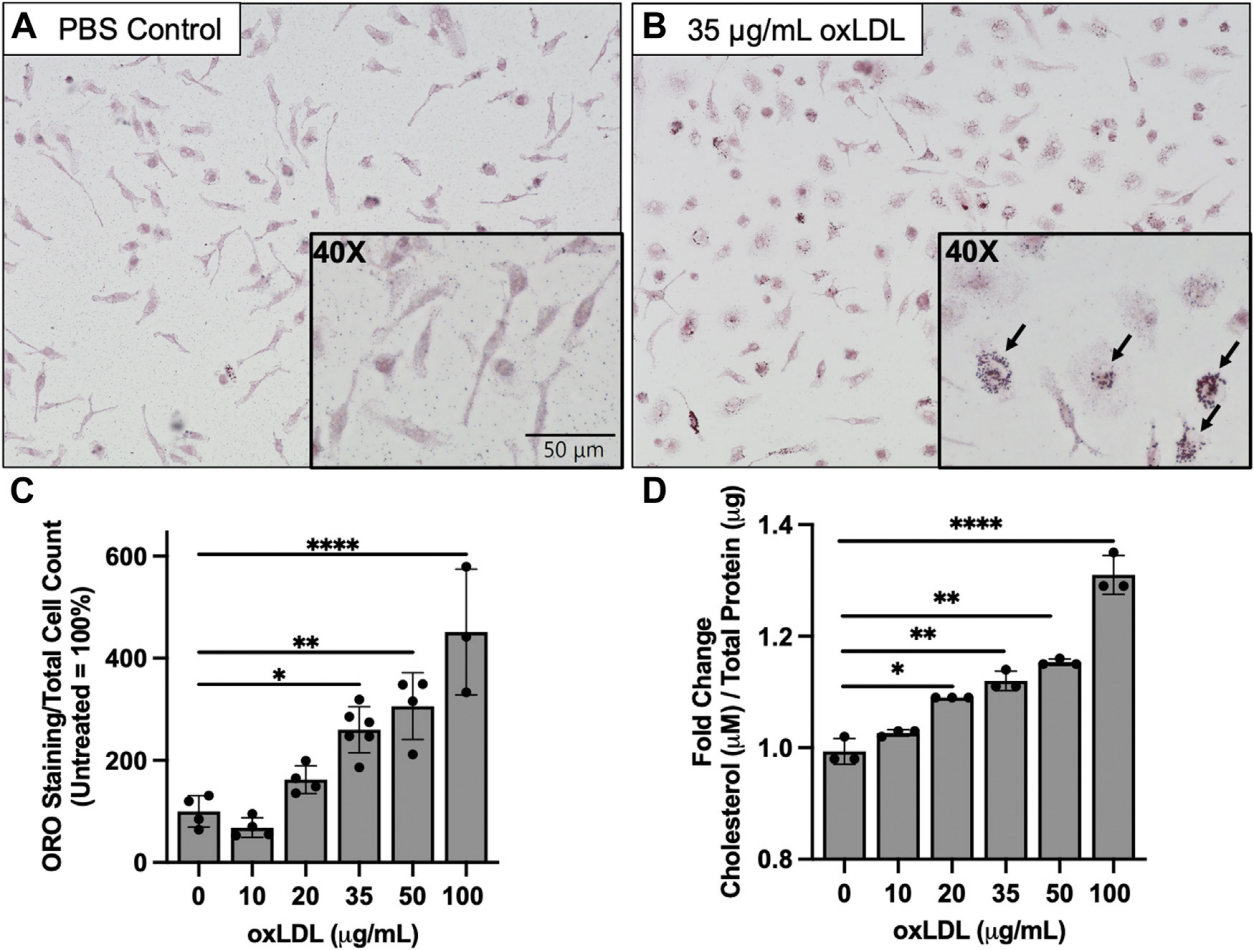
**Incubation of peritoneal macrophages with oxLDL generates foam cells.** Murine peritoneal macrophages from WT mice were incubated with increasing concentrations of oxLDL for 24 h and analyzed for foam cell formation and cholesterol accumulation. *A*, PBS-treated and *B*, 35 μg/ml oxLDL-treated macrophages were analyzed for lipid accumulation *via* ORO staining. Representative 20x images are shown with 40x images inserted into the *right corner*. *Arrows* indicate macrophage foam cells. *C*, images were quantified using ImageJ by dividing the area of staining by the total number of cells. Each data point represents an independent macrophage isolation. *D*, Amplex Red Cholesterol Assay kits were used to quantify the fold change of total cholesterol content. All data shown represent the mean ± SD (n = 3–6). Statistical analyses were determined using two-way ANOVA comparing each treatment condition with the PBS control group, ∗*p* < 0.05, ∗∗*p* < 0.01, ∗∗∗*p* < 0.001, and ∗∗∗∗*p* < 0.0001. ORO, Oil Red O; oxLDL, oxidized low-density lipoprotein.

To generate macrophage foam cells, primary peritoneal macrophages from WT mice were incubated with oxLDL particles for 24 h. Foam cells were visualized by staining macrophages with Oil Red O (ORO) following oxLDL treatments (Fig. 1, *A* and *B*). Foam cells were quantified by dividing the area of the ORO stain by total cell number (Fig. 1*C*). It was determined that a 24-h incubation period with 20 or 35 μg/ml oxLDL reproducibly generated quantifiable foam cells in peritoneal macrophages. Additionally, cholesterol concentrations increased with oxLDL treatment (Fig. 1*D*). As previously established (10, 27, 28, 29), these data demonstrate that primary macrophages incubated with oxLDL provides a good model system for studying foam cell formation.

### FFAR4 activation does not reduce macrophage foam cell formation

**Figure 2:**
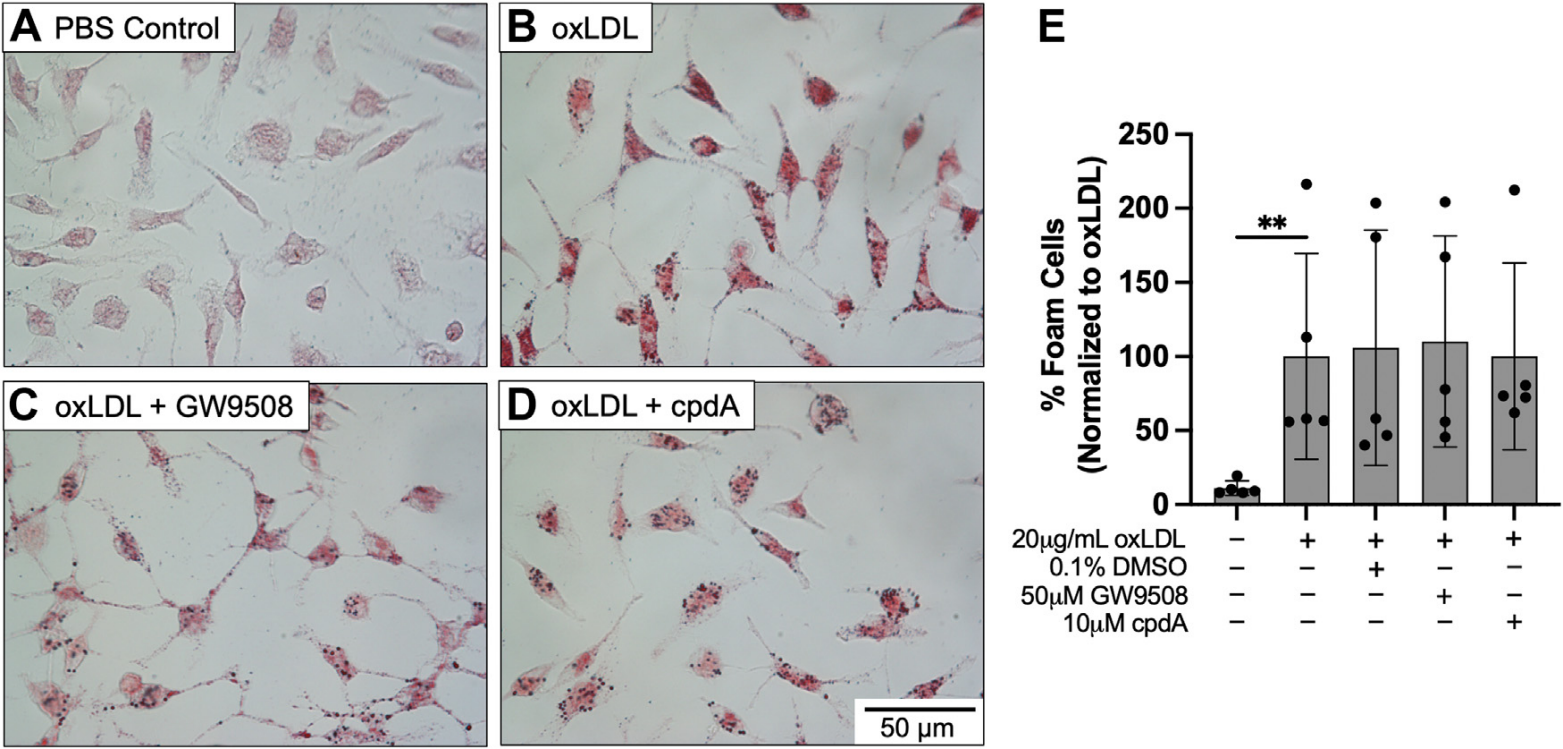
**FFAR4 activation does not reduce macrophage foam cell formation.** Peritoneal macrophages from WT mice were incubated with *A*, 0.1% dimethyl sulfoxide, *B*, 20 μg/ml oxLDL, *C*, 20 μg/ml oxLDL + 50 μM GW9508, or *D*, 20 μg/ml oxLDL + 10 μM cpdA for 24 h. Following treatment, macrophages were analyzed for foam cell formation *via* ORO staining (40x magnification). *E*, the number of foam cells were counted to determine a % foam cell for each condition. Each data point represents an independent macrophage isolation. Values represent the mean ± SD (n = 5). Statistics were calculated using a one-way ANOVA comparing the mean of each treatment to the oxLDL-treated control; ∗∗*p* < 0.01. FFAR, free fatty acid receptor; ORO, Oil Red O; oxLDL, oxidized low-density lipoprotein.

To test the effects of FFAR4 activation on macrophage foam cell formation, peritoneal macrophages were treated with 20 μg/ml oxLDL ± 0.1% dimethyl sulfoxide (DMSO), 50 μM GW9508, or 10 μM cpdA for 24 h. ORO staining of the cells showed that there were no differences in the number of foam cells when treated with FFAR4 agonists (Fig. 2). These data suggest that FFAR4 activation does not influence foam cell formation in peritoneal macrophages.

### Neither FFAR4 activation nor FFAR4 deficiency decrease cholesterol accumulation in oxLDL-treated macrophages

**Figure 3:**
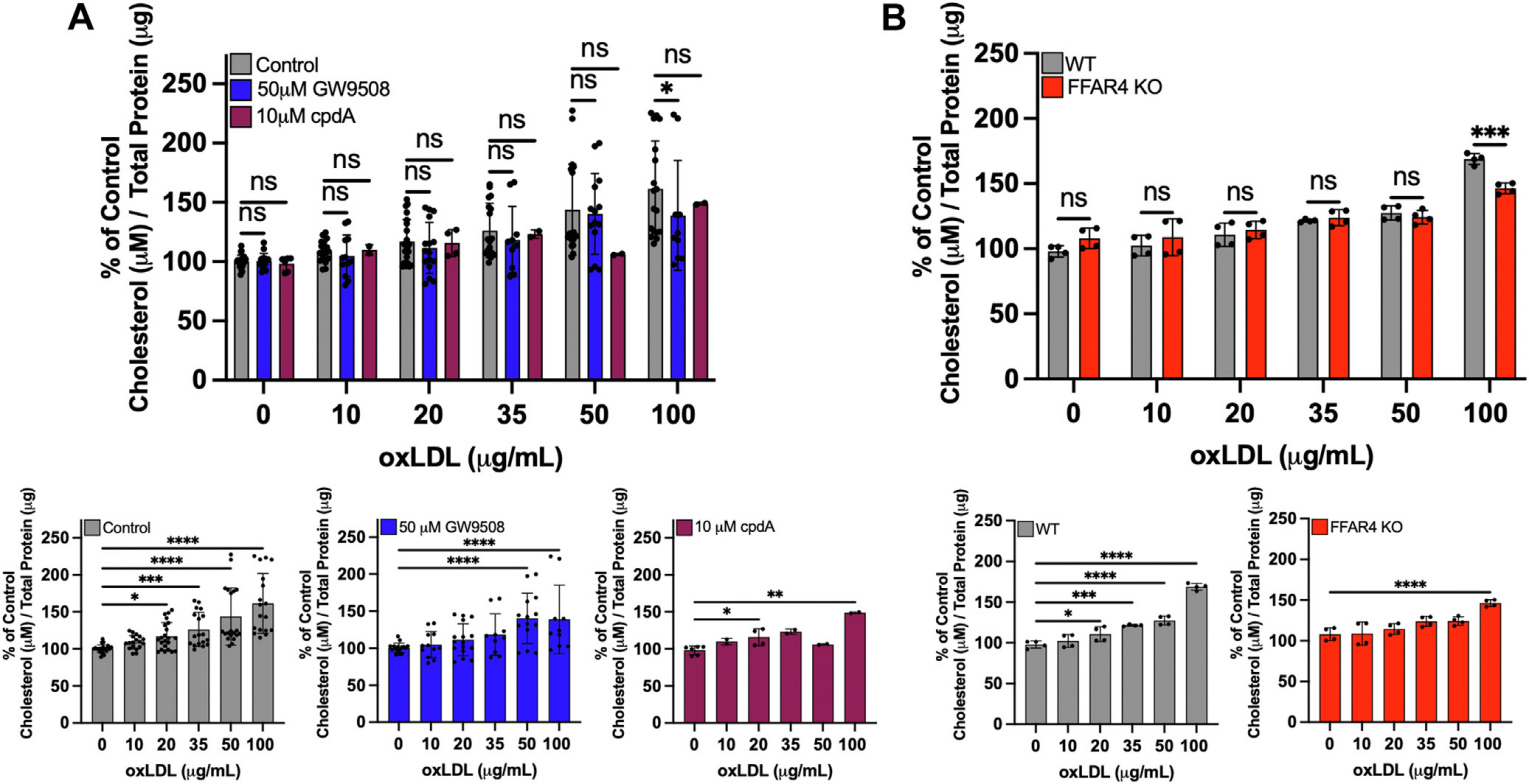
**Cellular cholesterol levels remain unchanged with FFAR4 activation or FFAR4 deficiency in oxLDL-treated macrophages.** Peritoneal macrophages were treated with 0 to 100 μg/ml oxLDL ± 0.1% dimethyl sulfoxide (*gray control*), 50 μM GW9508 (*blue*), or 10 μM cpdA (*maroon*) for 24 h. Intracellular cholesterol levels were quantified using AMPLEX Red cholesterol kits and represented as a percent of untreated controls. *A*, WT macrophages treated with FFAR4 agonist and *B*, WT (*gray*) and FFAR4 KO (*red*) oxLDL-treated macrophages are shown. Below *panel A*, data are separated and replotted to show changes in cholesterol concentration for each individual agonist. Below *panel B*, data are separated and replotted to show changes in cholesterol concentration for WT and FFAR4 KO macrophages. Values represent the mean ± SD. For *panel A*, the number of independent isolations ranged from 2 to 10 depending on the treatment, with 1 to 3 technical replicates per experiment. For *panel B*, we performed four independent isolations of WT and FFAR4 KO macrophages, with two replicates per experiment. Each independent isolation is a biological replicate and was the result of macrophages being pooled from 3 to 5 mice at a time. Statistics were calculated using a two-way ANOVA mixed model Bonferroni multiple comparisons test. ns = not significant, ∗*p* < 0.05, ∗∗*p* < 0.01, ∗∗∗*p* < 0.001, and ∗∗∗∗*p* < 0.0001. FFAR, free fatty acid receptor; oxLDL, oxidized low-density lipoprotein.

To further investigate if FFAR4 plays a role in macrophage foam cell formation, peritoneal macrophages were treated with 0 to 100 μg/ml oxLDL ± 0.1% DMSO, 50 μM GW9508, or 10 μM cpdA for 24 h and analyzed for total cholesterol levels. Neither activation of FFAR4 by GW9508 nor cpdA altered the cholesterol accumulated in the cells across all concentrations of oxLDL treatment (Fig. 3*A*). In parallel, we harvested peritoneal macrophages from WT and *Ffar4*^*−/−*^ mice and, when incubated with increasing concentrations of oxLDL, no differences in cellular cholesterol levels were observed (Fig. 3*B*). These data further support that FFAR4 does not play an important role in macrophage foam cell formation.

### Neither FFAR4 activation nor FFAR4 deficiency alter the uptake of Dil-oxLDL into macrophages

**Figure 4:**
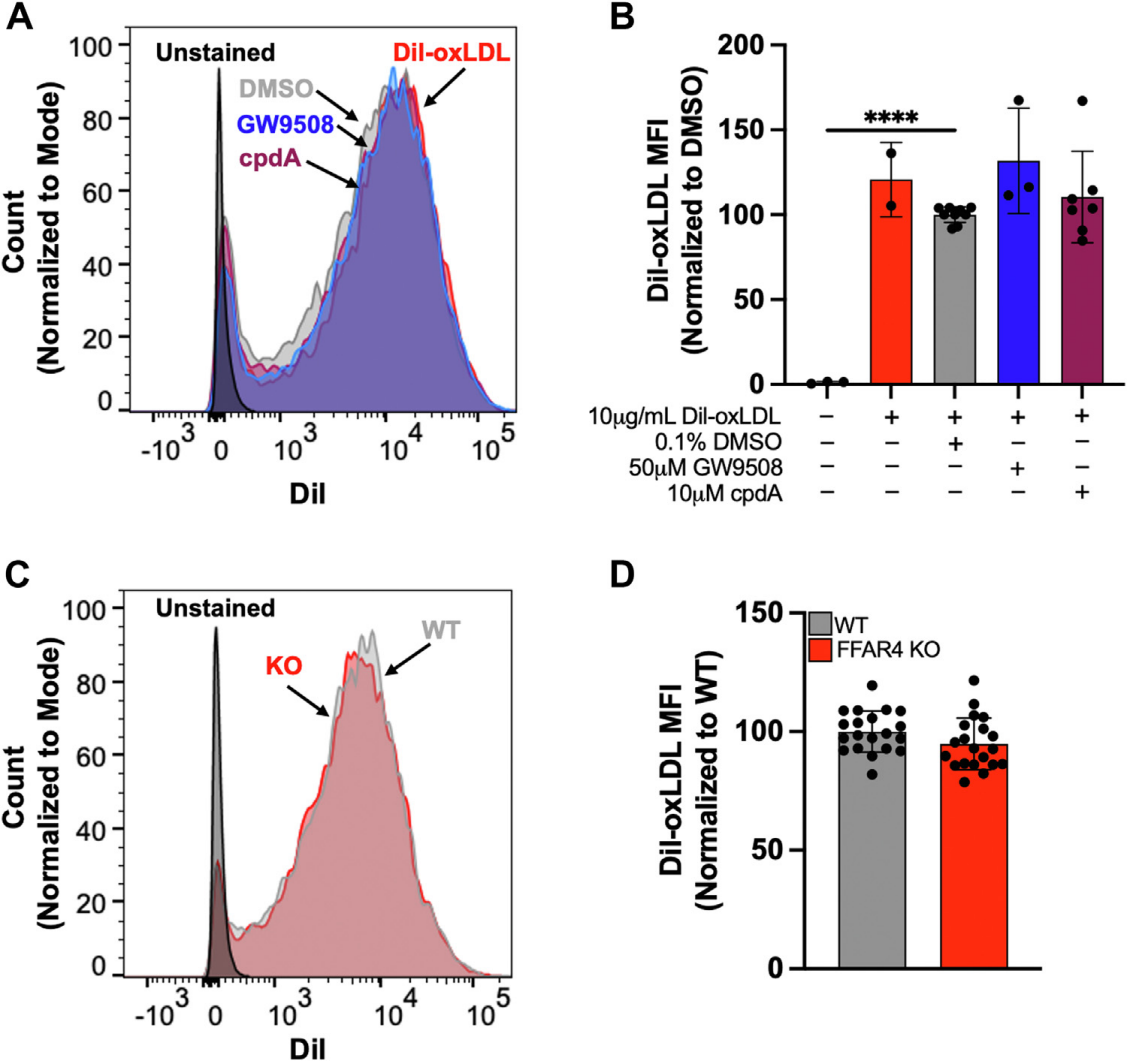
**Neither FFAR4 activation nor FFAR4 deficiency alters the uptake of Dil-oxLDL into macrophages.** Peritoneal macrophages were treated with 10 μg/ml Dil-oxLDL and 0.1% DMSO (*gray*), 50 μM GW9508 (*blue*), or 10 μM cpdA (*maroon*) for 2 h. Dil-oxLDL uptake was quantified *via* flow cytometry. *A*, histograms of WT macrophages treated with Dil-oxLDL and FFAR4 agonist. *B*, quantified Dil geometric mean fluorescence intensity (MFI) values. *C*, histograms of WT (*gray*) and FFAR4 KO (*red*) Dil-oxLDL–treated macrophages. *D*, quantified Dil geometric MFI values. Values represent the mean ± SD. For *panels A* and *B*, we performed three independent isolations with 1 to 3 replicates per experiment. For *panels C and D*, we performed five independent isolations of WT and FFAR4 KO macrophages, with 1 to 3 replicates per experiment. Values represent the mean ± SD. Statistics for WT macrophages treated with Dil-oxLDL and FFAR4 agonist were calculated using a one-way ANOVA comparing the mean of each treatment to the Dil-oxLDL–treated control. For WT and FFAR4 KO macrophages treated with Dil-oxLDL, statistics were calculated using unpaired *t*-tests, ∗∗∗∗*p* < 0.0001. DIL-oxLDL, 1,1′-dioctadecyl-3,3,3′,3′-tetramethylindocyanide perchlorate oxLDL; FFAR, free fatty acid receptor; oxLDL, oxidized low-density lipoprotein.

To investigate if FFAR4 influences the uptake of oxLDL in macrophages, cells were incubated with 10 μg/ml 1,1′-dioctadecyl-3,3,3′,3′-tetramethylindocyanide perchlorate oxLDL (DIL-oxLDL) ± 0.1% DMSO, 50 μM GW9508, or 10 μM cpdA for 2 h and DiI fluorescence was analyzed *via* flow cytometry. Uptake of Dil-oxLDL was unaffected by treatment with either of the FFAR4 agonists (Fig. 4, *A* and *B*). Additionally, there were no differences in the uptake of Dil-oxLDL in WT and FFAR4 KO macrophages (Fig. 4, *C* and *D*). These data demonstrate that FFAR4 does not influence the uptake of oxLDL into macrophages.

## Discussion

The literature suggests that activation of macrophage FFAR4 by synthetic agonists reduces inflammation, promotes cholesterol efflux, and stimulates macrophage migration (21, 23, 24). Additionally, *in vivo* studies have shown that activation of FFAR4 by GW9508 or TUG-891 reduces the size of atherosclerotic plaques in apoE KO mice (21, 22, 25). Together, these studies support the idea that FFAR4 may reduce atherosclerotic plaque formation by regulating inflammation and macrophage foam cell formation. The hallmark of early atherosclerosis is foam cell formation that results from an imbalance of cholesterol homeostasis (30). In this study, we used WT and FFAR4^−/−^ peritoneal macrophages to test the hypothesis that FFAR4 can protect against macrophage foam cell formation.

While FFAR4 seemed to be a novel target to prevent macrophage foam cell formation, our data provide no evidence that FFAR4 activation or FFAR4 deficiency impacts the foam cell outcome in oxLDL-treated macrophages. Using two different FFAR4 agonists, GW9508 and cpdA, we found that activation of the receptor did not protect against foam cell formation when macrophages were treated with oxLDL (Fig. 2). Further, activation of FFAR4 did not reduce the increased levels of cholesterol that resulted from oxLDL treatment of the cells (Fig. 3*A*). We also utilized FFAR4 KO mice to determine if loss of the receptor would have any impact on cellular cholesterol levels, but again observed no differences between WT and FFAR4 KO macrophages treated with increasing concentrations of oxLDL (Fig. 3*B*). Lastly, we observed that neither activation (Fig. 4, *A* and *B*) nor loss of FFAR4 (Fig. 4, *C* and *D*) altered the uptake of Dil-oxLDL into the macrophage. Taken together, these data suggest that FFAR4 does not play a role in dictating the outcome of foam cell formation in oxLDL-treated macrophages.

In this study, our hypothesis was shown to be incorrect, suggesting that the cardioprotective effects of FFAR4 in atherosclerosis are independent of lipoprotein uptake and foam cell formation. Previously, it has been shown that FFAR4 is an important inflammation regulator in macrophages (21). Additionally, activation of FFAR4 in mice shifted lesion macrophages toward a more anti-inflammatory phenotype and reduced atherosclerotic plaque development (25, 26). Inflammation is an essential contributor to the progression of atherosclerotic plaque development as it controls the recruitment, retention, and polarization of immune cells in the growing lesion (31). Additionally, lipid accumulation in macrophage foam cells is a balance between the uptake of oxLDL and the efflux of free cholesterol (30). Others have shown that FFAR4 activation helps facilitate cholesterol efflux from macrophages (23). It is possible that the receptor does not impact the uptake of lipids but rather helps promote the removal of excess lipids from the cell. Our studies did not specifically test the efflux capacity of the cells as HDL was not added during the cellular treatments. Both the regulation of lesion inflammation and the promotion of cholesterol efflux could explain the protective role FFAR4 plays in CVD. Taken together, FFAR4 remains a promising target for CVD protection, but additional studies are needed to fully understand the FFAR4-dependent mechanisms that afford CVD protection.

## Experimental procedures

### Materials

Ammonium-chloride-potassium lysis buffer, RPMI 1640 media, AMPLEX red cholesterol kits, Prolong Gold Antifade Mountant with DNA Stain 4′,6-diamidino-2-phenylindole, Dil-oxLDL, and thioglycolate medium were purchased from Thermo Fisher Scientific. ORO was from Sigma-Aldrich. Human oxLDL was from Lee Biosolutions, Inc. All other reagents were of analytical grade.

### Preparation of FFAR4 agonists

FFAR4 agonists GW9508 (10008907) and cpdA (16624) were purchased from Cayman Chemicals as a powder. Ligands were resuspended in DMSO at a final concentration of 10 mM. For experimental procedures, 50 μM GW9508 and 10 μM cpdA were used as these concentrations were determined to be sufficient to activate the receptor and cause cellular responses, while not being toxic to macrophages.

### Animal care

Experimental procedures in mice performed in this study conformed to the Guide for the Care and Use of Laboratory Animals (NIH publication, eighth Edition, 2011) and were approved by the Institutional Animal Care and Use Committee of the Medical College of Wisconsin. All mice were housed and maintained under a normal light-dark cycle in a pathogen-free barrier facility in accordance with federal and institutional guidelines and fed a standard chow diet. *Ffar4*^*+/+*^ and global *Ffar4*-deficient mice (*Ffar4*^*−/−*^) mice on the *C57bl6/N* strain were generated by crossing FFAR4 heterozygote (*Ffar4*^*+/−*^) mice (kind gift from Dr Vincent Poitout, University of Montreal) (32).

### Isolation and maintenance of peritoneal macrophages

Peritoneal macrophages from male and female mice were isolated and maintained as previously described (33). Data from male and female mice were combined together in the article due to no variation in the results depending on the sex of the animal that the macrophages originated from.

### Isolation and oxidation of LDLs

Lipoproteins were isolated by density gradient ultracentrifugation (34) and LDLs were oxidized as previously described (33). In addition to making our own oxLDL, purchased oxLDL from Lee Biosolutions was occasionally used in experiments. Both sources of oxLDL consistently produced the same experimental results.

### Macrophage foam cell generation and quantification

To generate macrophage foam cells, peritoneal macrophages were seeded in a 6-well plate on a cover slip at a density of 0.75 × 10^6^ cells per well. The following day, the media was replaced with fresh media containing 0 to 100 μg/ml oxLDL ± 0.1% DMSO, 50 μM GW9508, or 10 μM cpdA, and incubated for 24 h at 37 ˚C/5% CO_2_. To visualize foam cells, cells were first washed 2x with PBS and then fixed in 4% paraformaldehyde at room temperature for 15 min. Cells were then treated with a 0.156% ORO working solution made fresh immediately before staining. The working ORO solution was created by adding 0.2% (w/v) ORO in 100% methanol “ORO stock solution” to 1 M NaOH at a 0.22:0.78 ratio of 1 M NaOH: stock ORO solution. The working solution was filtered through a 0.45 μm filter to remove any precipitated ORO and incubated on the cells for 5 min. Cells were then washed 2x with PBS and coverslips were mounted onto a glass slide containing Prolong Gold Antifade Mountant with DNA Stain 4′,6-diamidino-2-phenylindole and sealed with clear nail polish. The slides were imaged using a Keyence All-in-One Fluorescence Microscope (BZ-X800). For each slide, 3 to 6 20x images were randomly captured for quantification. Images were analyzed by calculating the % foams cells on each slide or by dividing the total ORO-stained area by the total number of foam cells per image using ImageJ Fiji software (NIH; https://imagej.net/downloads).

### Cell lysis and cholesterol quantification

Peritoneal macrophages were incubated with oxLDL ± 0.1% DMSO, 50 μM GW9508, or 10 μM cpdA for 24 h. Cell lysates were harvested from cells by incubating with radioimmunoprecipitation assay buffer containing protease inhibitors (aprotinin, leupeptin, PMSF, and pepstatin) on ice for 10 min. Lysates were clarified by centrifugation at 8,000×*g* for 10 min at 4 °C. Total cellular cholesterol concentrations were determined using AMPLEX red cholesterol kits following the manufacturer’s protocol. Total protein concentration of lysates was determined using the Lowry method (35).

### Dil-oxLDL uptake assay

Peritoneal macrophages were seeded at a density of 1.0 × 10^6^ cells per well in a 12-well plate. Cells were serum-starved in RPMI media containing 0.5% bovine serum albumin for 12 h. Cells were then incubated with 10 μg/ml Dil-oxLDL ± 0.1% DMSO, 50 μM GW9508, or 10 μM cpdA for 2 h at 37 ˚C and analyzed for uptake in Dil-oxLDL using a FACSCelesta flow cytometer (BD Biosciences; Versiti Blood Center of Wisconsin) and FACSDiva software (https://www.bdbiosciences.com/en-us/products/software/instrument-software/bd-facsdiva-software). Flowjo software version 10.8.1 (Tree Star; https://www.flowjo.com) was used to analyze the data using geometric mean fluorescence intensity to compare samples.

### Statistics

Each experiment was repeated at least three times, with macrophages harvested from three independent isolations. Statistical analyses were performed using GraphPad Prism version 10.3.0 (https://www.graphpad.com), with details of each analysis provided in the Figure legends.

## Data availability

All data are present in the article.

## Supporting information

This article contains supporting information (33, 34, 36).

## Conflict of interest

The authors declare that they have no conflicts of interest with the contents of this article.
