# Supplementary material for: The cardioprotective role of the G protein–coupled receptor FFAR4 in atherosclerosis is independent of macrophage foam cell regulation
Source: J Biol Chem. 2025 Mar 27;301(5):108463. doi: 10.1016/j.jbc.2025.108463 (PMC12147181; doi:10.1016/j.jbc.2025.108463)
Supplement: Supplementary Materials [file mmc1.docx]

**SUPPORTING INFORMATION**

**The Cardioprotective Role of the G Protein-Coupled Receptor FFAR4 in Atherosclerosis is Independent of Macrophage Foam Cell Regulation**

Gage M. Stuttgen^1,2,3^ Caroline J. Ring^4^, Vishnu S. Guda^4^, Guadalupe K. Valdivia Esparza^1,2^, and Daisy Sahoo*^1,2,3,4^

^1^Department of Biochemistry, Medical College of Wisconsin, Milwaukee, Wisconsin, USA

^2^Cardiovascular Center, Medical College of Wisconsin, Milwaukee, Wisconsin, USA

^3^Center for Immunology, Medical College of Wisconsin, Milwaukee, Wisconsin, USA

^4^Department of Medicine, Division of Endocrinology & Molecular Medicine, Medical College of Wisconsin, Milwaukee, Wisconsin, USA

**COMPREHENSIVE MATERIAL AND METHODS**

*RNA Extraction and Gene Expression Analyses Using qPCR*

Total RNA was extracted using the Total RNA Purification Kit (Norgen Biotek Corp, 69504) following the manufacturer’s instructions. Contaminating DNA was digested with DNase I (ThermoFisher, EN0521) before 1 µg total RNA was reverse-transcribed into single-stranded cDNA using the high-capacity cDNA reverse transcriptase kit (ThermoFisher, 4368814). RNA concentrations (ng/µL), A260/280, and A260/230 ratios were measured using an ND-1000 Spectrophotometer (Nanodrop, Wilmington, DE, USA). Thermal Cycler Quantitative PCR was performed using SYBR Green on a 384-well Bio-Rad PCR Detection System (Hercules, CA, USA) with a 40-cycle threshold limit. Relative gene expression was measured by the 2^-(ΔΔCt)^ method described by the three equations below using the β-actin as a reference gene. Controls (no reverse transcriptase) and melt curves were used to determine sample genomic DNA contamination and PCR product specificity, respectively. Primer sequences used for qRT-PCR are listed in **Supplemental Table 1**. All primers used were validated to be efficient and specific for their gene target via standard curve analysis.

$$\boldsymbol{Equation 1:} Ct(sample)=Ct\left( target gene \right)-Ct(reference gene)$$

$$\boldsymbol{Equation 2:} Ct=Ct\left( experiment sample \right)-Ct(control sample)$$

$$\boldsymbol{Equation 3:}Fold change=2^{-Ct}$$

*HEK293 Cell Culture and Transfection*

HEK293 cells were maintained in Dulbecco’s Modified Eagle’s Medium (DMEM; Invitrogen 11965-092) supplemented with 2 mM L-glutamine, 50 units/mL penicillin, 50 μg/mL streptomycin, and 10% (v/v) fetal bovine serum (FBS) within a 37°C/5% CO_2_ cell culture incubator and passaged every 3-4 days. For transient transfections and subsequent experiments, approximately 2 x 10^6^ of cells were plated on 10-cm cell culture dishes and returned to the incubator. Upon reaching 70-90% confluency, HEK293 cells were transiently transfected with cDNA encoding empty pCMV6 plasmid vector or vectors containing FFAR4 cDNA using FuGENE 6 transfection agent (Promega, E2691), according to the manufacturer’s instructions.

*Seeding of HEK293 Cells for Experiments*

At 24 h post-transfection with empty vector or FFAR4, HEK293 cells were detached from 10-cm dishes by incubating in 1 mL 0.05% trypsin-ethylenediaminetetraacetic acid (EDTA) solution (Gibco) for 5-7 min at 37°C. Trypsin was neutralized by adding 5 mL complete DMEM, and the cell suspension was aliquoted into 6-well (1 mL/well), 12-well (0.4 mL/well), or 96-well (0.1 mL/well) cell culture plates for technical replicates and/or separate experiments. All experiments were performed at 48 h post-transfection of HEK293 cells, unless otherwise stated.

*Calcium Flux Experiments*

To perform calcium flux experiments FFAR4-transfected HEK293 cells were plated at a density of 50,000 cells/well in 96-well plates. Cells were washed with calcium/magnesium-free HBSS supplemented with 20mM HEPES (Thermo Fisher Scientific, Waltham, MA) and 0.1% (v/v) BSA Fraction V (Gibco, 15260-037) and loaded with Calcium 6 dye (Molecular Devices, 8190) for 1h at 37°C and 5% CO_2_. FFAR4 agonists (GW9508 and cpdA) were diluted in calcium/magnesium-free HBSS buffered with 20mM HEPES and were loaded onto a separate compound plate. Fluorescence was measured at 37°C with a FlexStation3 Multimode Microplate Reader (Molecular Devices) with excitation and emission wavelengths of 485nm and 515nm, respectively. FFAR4 agonists were resuspended at the concentrations indicated in the figure legends and added to the cells after baseline fluorescence was measured for 20 sec. The percentage Ca^+2^ flux was calculated from the maximum fluorescence minus the minimum fluorescence as a percent of baseline fluorescence. EC_50_ values were determined by nonlinear fitting to a four-parameter logistic function.

*Immunoblot Analyses*

Cellular lysates (10-20 µg) were combined with an equal volume of 2X sample treatment buffer (STB) (4% SDS, 20% glycerol, 120 mM Tris-HCL [pH 6.8], 5% β-mercaptoethanol [BME], bromophenol blue) and boiled for 95°C for 5 min. Samples were then separated by 10% SDS-PAGE at constant voltage (100V) and wet-transferred to a nitrocellulose membrane. The membranes were blocked in 5% milk in tris-buffered saline with tween (TBST) at room temperature and then incubated with the primary antibody in 1% milk overnight at 4°C. Membranes were washed 3x in TBST at room temperature and then incubated with horseradish peroxidase-conjugated secondary antibody in 1% milk at room temperature for 1 h. SuperSignal West Pico (34580) or Femto (34094) Chemiluminescent Substrate (Thermofisher) was allowed to react with membranes for 5 min before blots were imaged using a ChemiDoc MP or x-ray film. The intensity/optical density values of the bands were measured using Bio-Rad Image Lab Software or NIH FIJI/ImageJ Software and normalized to a housekeeping gene (β-actin). Antibodies used for immunoblot analyses and their respective dilutions are listed in **Supplemental Table 2.**

*SYTOX Cell Death Assay*

Macrophages were plated in a black flat bottom 96-well plate (CLS3603) at a density of 0.1 x 10^6^ cells per well. Treatments were performed on the cells for 24 h using 100 µL volumes. To assess cell death, SYTOX (S7020) from Thermo Fisher Scientific (Waltham, MA) was added to each well following the manufacturing protocol. Briefly, 25 µL of 50 µM SYTOX was added directly to the treatment media, so the final concentration was 10 µM in each well. To determine a maximum cell death, some cells were treated with 120 µM digitonin during the SYTOX incubation. After the SYTOX was added to the plate, the plate was incubated at 37°C for 30 min in the plate reader prior to reading the fluorescence at 504/523nm. Cell death was calculated using the following formula:

$$\% cell death=\left( \frac{Flourescence of treatment}{Flourescence of maxiumum cell death} \right) x 100$$

*Lipoprotein Isolation*

Lipoproteins were isolated as previously described (1). Plasma was filtered through a 0.45 μm syringe filter and its density was adjusted to 1.025 g/mL with KBr. The sample was then overlayed with 1.019 g/mL saline buffer, centrifuged at 40,000 RPM (Beckman Ultracentrifuge, SW-40Ti rotor) for 24 h at 4°C. The resultant superior layer of very low-density lipoprotein (VLDL) was isolated, and the density of the remaining sample was adjusted to 1.080 g/mL with KBr, overlayed with 1.063 g/mL saline, centrifuged, and the resultant superior layer of low-density lipoprotein (LDL) was collected. Lipoprotein samples were dialyzed 4x against 4 L of PBS buffer. Lipoprotein purity was verified by FPLC analysis.

*Generation of oxLDL*

To generate oxLDL, LDL was oxidized by dialysis against 5 µM CuSO_4_ in PBS for 6 h at 37°C, and the reaction was stopped by dialysis in PBS containing 0.02% (0.54 nM) EDTA overnight at 4°C as previously described (2). An additional dialysis against PBS for 6 h at 4°C was performed to remove any remaining EDTA. Thiobarbituric acid reactive substance (TBAR) and electrophoretic mobility shift (EMSA) assays were performed to verify oxidation levels and changes in charge, respectively (3).

*Data Normalization and Statistical Analyses*

All statistical analyses were completed using GraphPad Prism 10.3.0 (San Diego, CA, USA). P-values were calculated by one-way analysis of variance (ANOVA), two-way ANOVA, or unpaired t-tests when appropriate, with details of each analysis provided in the figure legends. Multiple comparisons tests were performed as indicated for each experiment. Data values normalized to WT or unstimulated controls as a percentage (normalized value = 100%) or fold change (normalized value = 1). Data is presented as mean ± SD or mean ± SEM as indicated in the figure legends. For all statistics, ns means not significant, *P<0.05, **P<0.01, ***P<0.001, and ****P<0.0001.

**TABLES**

| **Gene** | **Forward Primer (5’ 🡪 3’)** | **Reverse Primer (5’ 🡪 3’)** |
| --- | --- | --- |
| *β-actin | CTCTGGCTCCTAGCACCATGAAGA | GTAAAACGCAGCTCAGTAACAGTCCG |
| FFAR4 | TCAGGCGAAATGACTTGTCTG | GCTGTTGGAAGTCGGGTATT |

##

## Supplemental Table 1: List of primers used for PCR analyses.

Reference genes indicated with an asterisk (*)

| **Antibody** | **Dilution (v/v)** | **Company** |
| --- | --- | --- |
| *Donkey-anti-rabbit-IgG-HRP | 1:5,000 – 1:10,000 | Amersham (NA934) |
| *Sheep-anti-mouse-IgG-HRP | 1:5,000 – 1:10,000 | Amersham (NA931) |
| Mouse-anti-β-actin | 1:5000 | Ambion Life Tech (AM4302) |
| Rabbit-anti-ERK1/2 | 1:1000 | Cell Signaling (4695) |
| Rabbit-anti-pERK1/2 | 1:1000 | Cell Signaling (4377) |

##

## Supplemental Table 2: Antibodies used for immunoblot analyses and their dilutions

Secondary antibodies are indicated with an asterisk (*).

**
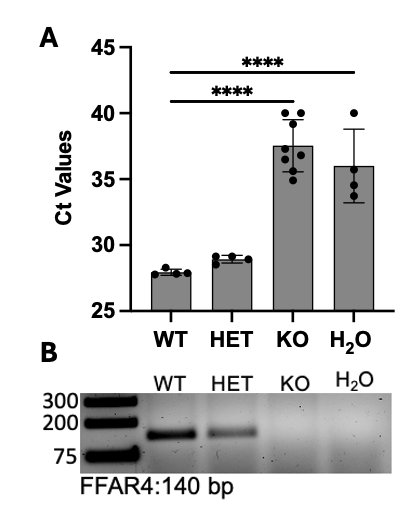
**

**Supplemental Figure 1: FFAR4 is expressed in WT peritoneal macrophages*.*** Peritoneal macrophages from wild-type (WT), FFAR4 heterozygous (HET), and FFAR4 knock-out (KO) mice were analyzed for FFAR4 RNA expression. Ct values from qRT-PCR reaction **(A).** Agarose gel showing DNA product from PCR reaction **(B).** H_2_O serves as a negative control in this experiment as it did not have any RNA in the PCR reaction.

**
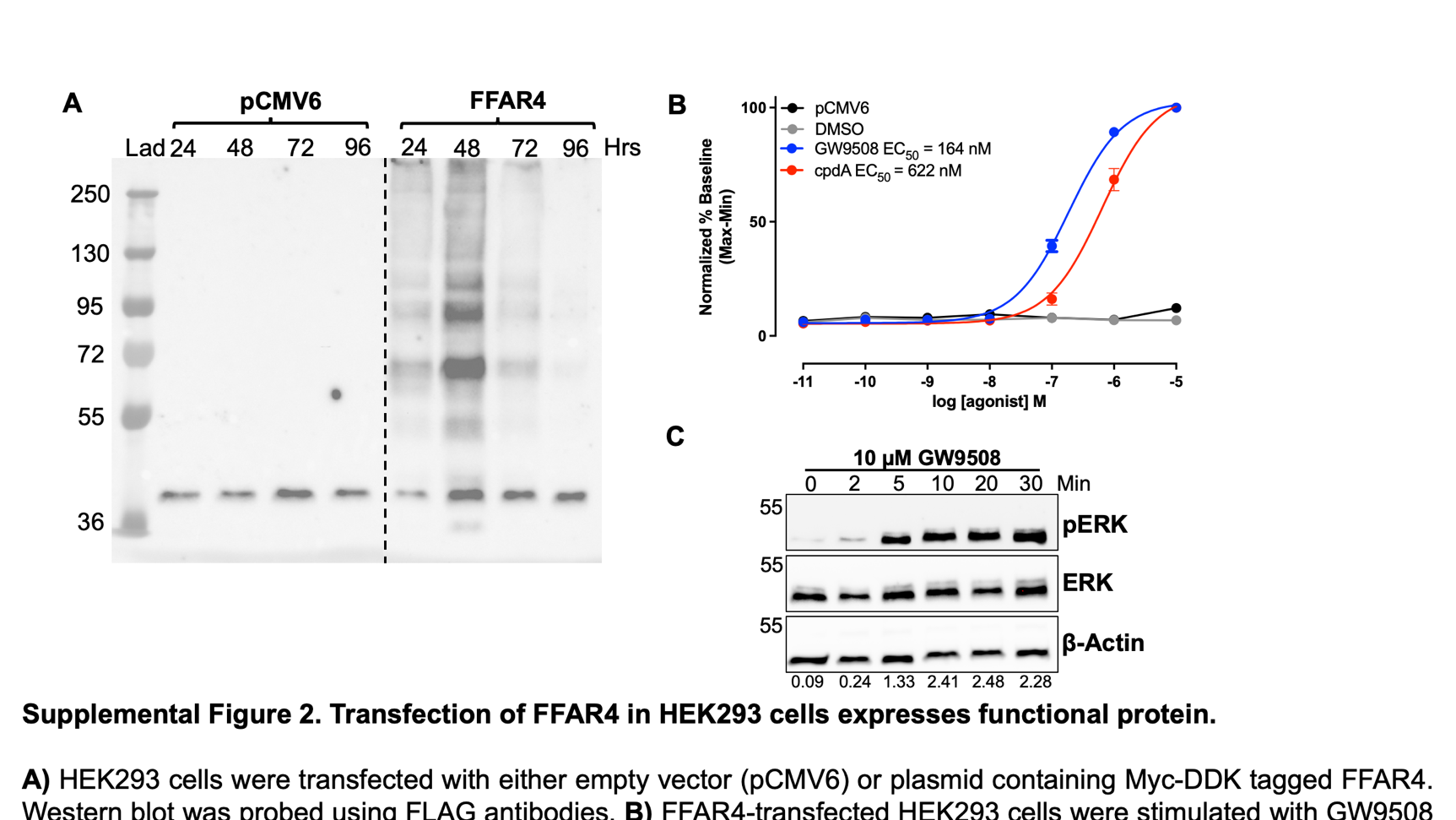
**

**Supplemental Figure 2: Transfection of FFAR4 cDNA in HEK293 cells results in expression of functional protein.** **A)** HEK293 cells were transfected with empty vector (pCMV6) or plasmid containing Myc-DDK-tagged murine FFAR4. The immunoblot was probed using FLAG antibodies. **B)** FFAR4-transfected HEK293 cells were stimulated with GW9508 or cpdA agonists (100µM-10pM) and calcium signaling was measured using Calcium 6 Dye. DMSO solvent control and pCMV6 empty vector control showed no calcium signaling. Data represent samples performed in duplicate and error bars represent standard deviation between replicates (n=3). **C)** FFAR4-transfected HEK293 cells were stimulated with 10µM GW9508 for 0-30min and analyzed for ERK signaling. Densitometry values of pERK/ERK ratios normalized to β-actin are shown below the lanes for each sample. Representative blots are shown for data (n=3, independent transfections).


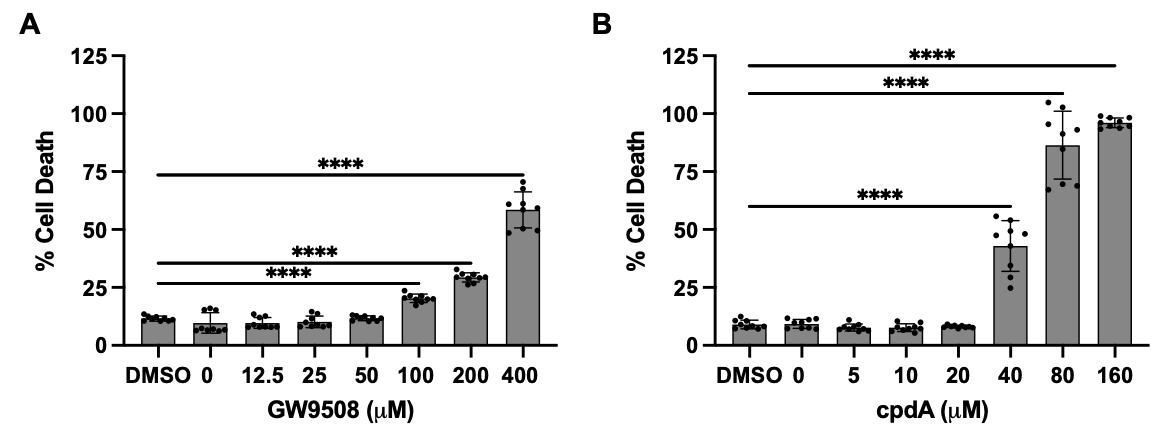
**Supplemental Figure 3: Determining FFAR4 agonist concentrations that are toxic to macrophages.** To test if GW9508 or cpdA is toxic to macrophages, peritoneal macrophages were incubated with increasing concentrations of **A)** GW9508 (0-400µM) or **B)** cpdA (0-160µM) for 24h and analyzed for cell death via SYTOX assays. All data shown represent the mean ± SD (N=3, in triplicate). Statistical analyses were determined using 2-way ANOVA comparing each treatment condition with the DMSO control group, ****P<0.0001.


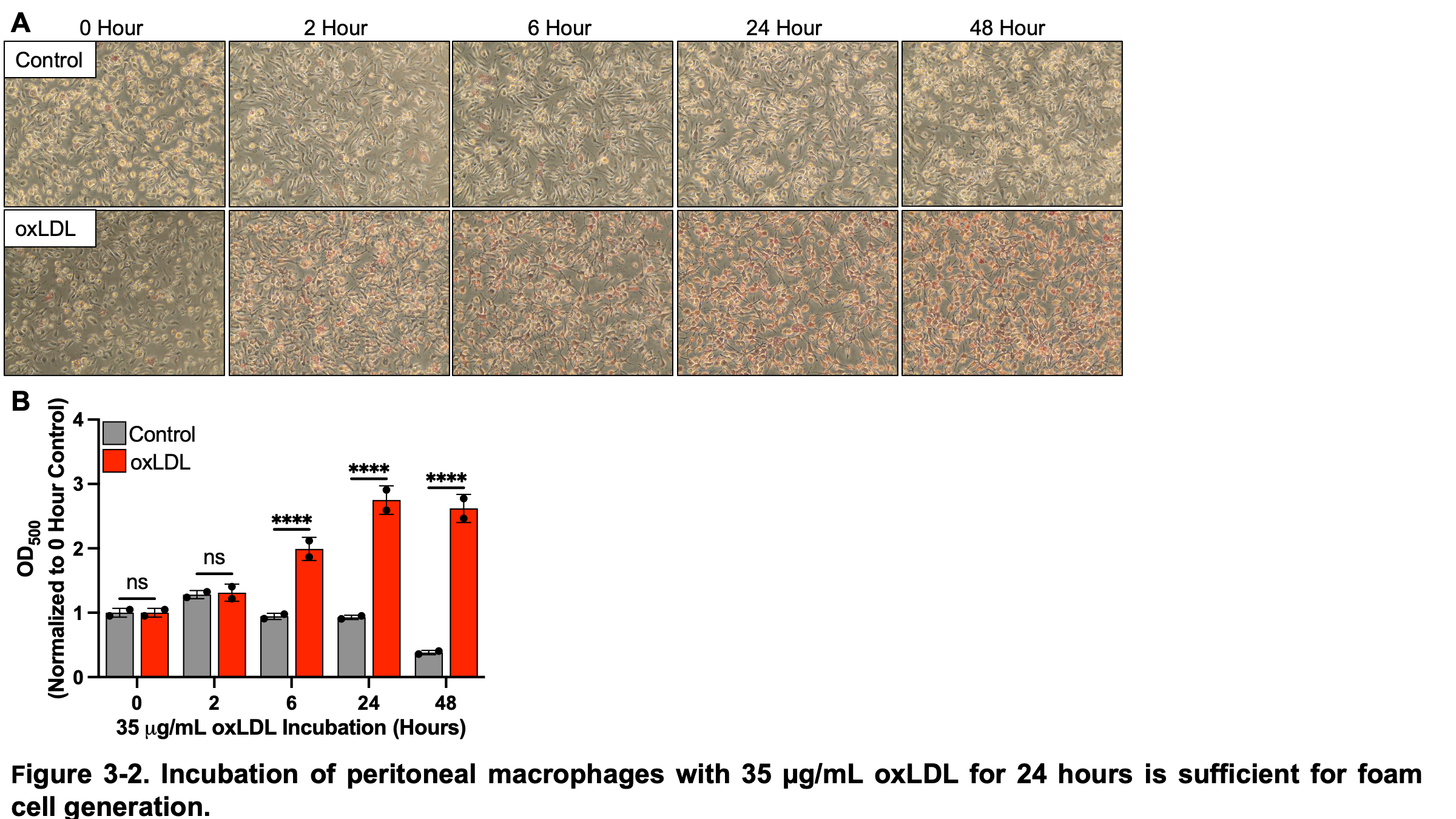


**Supplemental Figure 4: Time curve for foam cell formation.** Peritoneal macrophages were incubated with 35 µg/mL oxLDL for 0, 2, 6, 24, and 48 h. **(A)** Foam cells were visualized for lipid accumulation via Oil Red O (ORO) stain. **(B)** Foam cells were quantified by extracting ORO dye from cells with isopropanol. The extracted ORO was quantified by measuring absorbance at 500 nm using a plate reader. Data represent the mean ± SD (n=2). Statistical analyses were determined using two-way ANOVA comparing each oxLDL treatment time point with the corresponding control group, ns = not significant, ****P<0.0001.

**REFERENCES**

1. Castleberry, M., Raby, C. A., Ifrim, A., Shibata, Y., Matsushita, S., Ugawa, S., Miura, Y., Hori, A., Miida, T., Linton, M. F., Michell, D. L., Tsujita, M., and Vickers, K. C. (2023) High-density lipoproteins mediate small RNA intercellular communication between dendritic cells and macrophages. *J. Lipid Res.* **64**, 100328

2. Nicholson, A. C., Frieda, S., Pearce, A., and Silverstein, R. L. (1995) Oxidized LDL Binds to CD36 on Human Monocyte-Derived Macrophages and Transfected Cell Lines. *Arter., Thromb., Vasc. Biol.* **15**, 269–275

3. Bobek, J. M., Stuttgen, G. M., and Sahoo, D. (2024) A comprehensive analysis of the role of native and modified HDL in ER stress in primary macrophages. *Front. Cardiovasc. Med.* **11**, 1448607
